# Leveraging continuous glucose monitoring as a catalyst for behaviour change: a scoping review

**DOI:** 10.1186/s12966-024-01622-6

**Published:** 2024-07-10

**Authors:** Michelle R. Jospe, Kelli M. Richardson, Ahlam A. Saleh, Lauren C. Bohlen, Jacob Crawshaw, Yue Liao, Kristin Konnyu, Susan M. Schembre

**Affiliations:** 1 Department of Oncology, Lombardi Comprehensive Cancer Center, Georgetown University, Washington DC, USA; 2 School of Nutritional Sciences and Wellness, College of Agriculture, Life and Environmental Sciences, University of Arizona, Tucson, AZ USA; 3 Arizona Health Sciences Library, University of Arizona, Tucson, AZ USA; 4 Center for Health Promotion and Health Equity, Department of Behavioral and Social Sciences, Brown University School of Public Health, Providence, RI USA; 5 Centre for Implementation Research, Ottawa Hospital Research Institute, Ottawa, ON Canada; 6 Department of Kinesiology, College of Nursing and Health Innovation, University of Texas at Arlington, Arlington, TX USA; 7 Health Services Research Unit, Institute of Applied Health Sciences, University of Aberdeen, Aberdeen, Scotland

**Keywords:** Continuous glucose monitoring, Blood glucose self-monitoring, Biomarkers, Feedback, Behaviour change

## Abstract

**Background:**

Amidst the escalating prevalence of glucose-related chronic diseases, the advancements, potential uses, and growing accessibility of continuous glucose monitors (CGM) have piqued the interest of healthcare providers, consumers, and health behaviour researchers. Yet, there is a paucity of literature characterising the use of CGM in behavioural intervention research. This scoping review aims to describe targeted populations, health behaviours, health-related outcomes, and CGM protocols in randomised controlled trials (RCTs) that employed CGM to support health behaviour change.

**Methods:**

We searched Ovid MEDLINE, Elsevier Embase, Cochrane Central Register of Controlled Trials, EBSCOhost PsycINFO, and ProQuest Dissertations & Theses Global from inception to January 2024 for RCTs of behavioural interventions conducted in adults that incorporated CGM-based biological feedback. Citation searching was also performed. The review protocol was registered (https://doi.org/10.17605/OSF.IO/SJREA).

**Findings:**

Collectively, 5389 citations were obtained from databases and citation searching, 3995 articles were screened, and 31 were deemed eligible and included in the review. Most studies (*n* = 20/31, 65%) included adults with type 2 diabetes and reported HbA1c as an outcome (*n* = 29/31, 94%). CGM was most commonly used in interventions to target changes in diet (*n* = 27/31, 87%) and/or physical activity (*n* = 16/31, 52%). 42% (*n* = 13/31) of studies provided prospective CGM-based guidance on diet or activity, while 61% (*n* = 19/31) included retrospective CGM-based guidance. CGM data was typically unblinded (*n* = 24/31, 77%) and CGM-based biological feedback was most often provided through the CGM and two-way communication (*n* = 12/31, 39%). Communication typically occurred in-person (*n* = 13/31, 42%) once per CGM wear (*n* = 13/31; 42%).

**Conclusions:**

This scoping review reveals a predominant focus on diabetes in CGM-based interventions, pointing out a research gap in its wider application for behaviour change. Future research should expand the evidence base to support the use of CGM as a behaviour change tool and establish best practices for its implementation.

**Trial registration:**

doi.org/10.17605/OSF.IO/SJREA.

## Introduction

Healthcare has seen significant advancements in the use of wearable biosensors for real-time monitoring of specific biological analytes [1]. Such technology opens the door to delivering more personalised and timely interventions, which are pillars of the precision health movement [2]. Precision health offers a plausibly more efficacious approach to traditional ‘one-size-fits-all’ public health interventions by delivering the right support, to the right individual, based on their biological, behavioural, psychological, and social determinants of health [3, 4]. While some limitations of precision health still need to be addressed, such as inequities in social, environmental and economic influences [5], providing timely feedback that is based on one’s biological state (“biological feedback”) has great potential to support changes in behaviours that meaningfully impact health-related outcomes [6].

Biological feedback is defined as “providing individuals with their biological data through direct communication (via an unblinded body-worn assessment device such as a heart rate monitor or a continuous glucose monitor [CGM]); or indirect communication (via health coaches, patient educators, or messaging systems) about biological data to support health behaviour change explicitly or implicitly for improving health-related outcomes” [7]. This form of feedback is distinct from the traditional mind–body technique of “biofeedback,” which provides feedback on one’s autonomic nervous system to treat health conditions [8, 9]. In our recent scoping review, we found over 750 randomised controlled trials (RCTs) that used biological feedback to support health behaviour change [6]. Results from our scoping review indicated that many of these interventions aimed to modify diet and physical activity behaviours based on data from glucose monitors, particularly among people with diabetes. Given the prevalence of interventions focusing on glucose monitoring, it is crucial to delve deeper into the role of biological feedback from CGMs, which are reshaping the way we understand and manage metabolic dysfunction.

In the rapidly evolving field of healthcare technologies, CGM stands out as particularly pivotal. In contrast to the intermittent data provided by traditional methods of self-monitoring of blood glucose with a glucometer, CGM offers the advantage of collecting real-time glucose data continuously, providing a comprehensive overview of glucose levels and trends. These data can be used to inform personalised behavioural and pharmacological interventions aimed at improving glycaemic control outcomes [10]. The significance of CGM is underscored by its dominance in the biosensor market [1]. CGM was initially introduced in 1999 as a diabetes management tool for people living with type 1 diabetes mellitus, reducing reliance on fingerpricks from glucometers [11]. Nearly a quarter-century later, CGM-based biological feedback is in use within a broader market, fuelling the rise of global digital health startups. These companies mainly target people without diabetes, people desiring weight loss, athletes, and health enthusiasts. Using advanced data analytics, individuals’ CGM data are integrated with their related behavioural, biological, and psychosocial data to offer real-time insights into how food, sleep, exercise, and stress impact their glucose trends with a goal of optimising health and performance.

Despite the increasing popularity of CGM as a health behaviour change tool, there is a paucity of literature characterising the use of CGM in behavioural intervention research [12, 13]. The use of CGM in research is diverse, with CGM wear periods ranging from a couple of days to several months, and includes variations in whether participants can view CGM data in real time, as well as differences in how this data is interpreted. This leaves a significant gap in the collective understanding of how wearable biosensors can be best employed to affect meaningful health behaviour change. As technology and healthcare continue to intersect, it is becoming increasingly essential to develop best practices that optimise the effectiveness of behavioural interventions leveraging these tools. Therefore, the objectives of this scoping review were to: (1) describe the patient populations, health behaviours, and health-related outcomes targeted by CGM-based biological feedback interventions, and (2) characterise the methods by which CGM is used as a behaviour change tool within RCTs aimed to support health behaviour change.

## Methods

### Overview

Our aims align with the indications for a scoping review, which include identifying what evidence is available and which knowledge gaps remain, investigating the methods of research conduct, and utilising the findings as precursor to the feasibility of a systematic review and meta-analysis; thus, justifying the scoping review approach [14]. The *Joanna Briggs Institute Reviewer Manual* [15] was used to guide the review methods. The Preferred Reporting Items for Systematic Reviews and Meta-Analyses Extension for Scoping Reviews (PRISMA-ScR) checklist was followed [16]. The review was registered in Open Science Framework Registries (10.17605/OSF.IO/SJREA) [17].

### Search strategy, selection criteria and review management

We collaborated with a research librarian to devise a search strategy based on our prior scoping review of 767 RCTs utilising biological feedback to support health behaviour change [6]. The prior search was conducted in June 2021 with no limitation of publication date. Here, relevant subject terms and text-words were included to capture behavioural interventions that incorporated feedback and biological measures, including glucose monitoring. For the current review, we updated the prior search and added terminology specific to CGM. The full search strategy has been included as Appendix 1. The updated search strategy was applied to articles published through January 2024, with no limit on year of publication. The search strategy was modified for the following electronic databases: Ovid MEDLINE, Elsevier Embase, Cochrane Central Register of Controlled Trials, EBSCOhost PsycINFO, and ProQuest Dissertations & Theses Global. Bibliographies of 17 additional reviews were also searched, and relevant articles were retained. There were no restrictions based on language.

Records returned by the search were deduplicated using EndNote 20 (Clarivate Analytics, Boston, MA) and added to the literature review software, DistillerSR® (Evidence Partners; Ottawa, Canada) for screening and data extraction. An additional deduplication process (using artificial intelligence) was applied in DistillerSR® to confirm all duplicate records were removed. Retracted articles were additionally identified using EndNote 20 and removed.

A multistep process was followed to determine study eligibility based on the following inclusion criteria: human adults ≥ 18 years, primary analyses of RCTs published in a peer-review journal or as a thesis or dissertation, and have at least one study arm receiving CGM-based biological feedback to support a health behaviour change. First, two trained reviewers completed an independent, single-entry title and abstract screening phase for initial eligibility. An artificial intelligence feature within DistillerSR® was used to confirm no abstracts were erroneously excluded. Then, full text versions of initially eligible articles were retrieved. Two trained reviewers completed a full text screening phase in which the preliminary inclusion criteria were confirmed and the use of CGM data to promote behaviour change was determined. If the use of CGM was unclear from the full text, an in-depth review of the study protocols available from trial registrations or published protocol was conducted. Articles not available in English were translated using Google Translate. Double-data entry by two independent reviewers for the full text screening phase was used for quality assurance. Conflicts were discussed between the two reviewers and resolved. If a conflict could not be resolved by the two reviewers, a third qualified reviewer made the final determination.

### Data extraction

Extracted data were selected based on the Taxonomy of Technology-Enabled Self-Management Interventions [18] and CGM-specific reporting guidelines by Wagner and colleagues [19]. Data were also consistent with the three active components of personalised interventions: (1) sensing, (2) reasoning, and (3) acting [20]. *Sensing* describes the input parameters (ie, glucose) needed for the personalised intervention and how the measurement is performed (ie, CGM) [20]. *Reasoning* refers to providing feedback that is based on the input data (ie, biological feedback), including personalised behaviour recommendations or disease management guidance. Lastly, *acting* refers to how the biological feedback is communicated to the consumer to promote behaviour change (e.g., the mode, channel, frequency, and timing) [20]. Based on these criteria, a data extraction form was developed within DistillerSR®. The data extraction form was piloted by the three reviewers and refined prior to use. Extraction items included bibliographic data, participant characteristics, study design, CGM characteristics and wear durations, and CGM use (Appendix 2). Information related to the study design and treatment of all study arms were extracted, for reference. Results of included RCTs were not extracted as a synthesis of findings was not the objective of our scoping review [14], hence a risk of bias assessment was not completed. Double-data extraction of the included full text articles was then performed by the two primary reviewers. When necessary and if available, previously published study protocols or protocol details from clinical trial registries were reviewed. Data that were unobtainable have been described as “unclear.” Conflicts were discussed between the primary reviewers and resolved. If a conflict could not be resolved, the third reviewer made the final determination. The extracted data in DistillerSR® was downloaded and cleaned in OpenRefine [21].

## Results

**Fig. 1:**
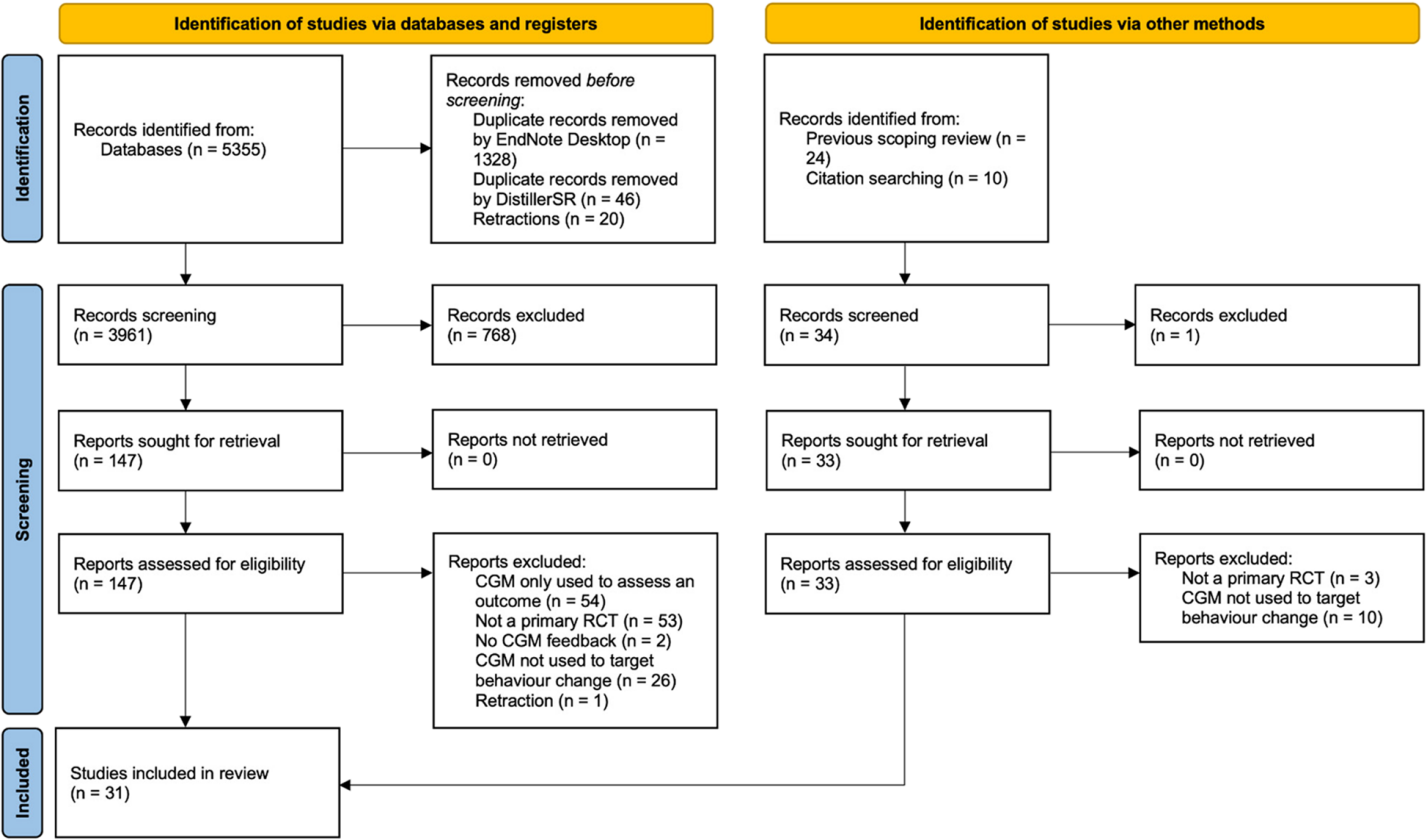
Preferred reporting items for systematic reviews and meta-analyses extension for scoping reviews (PRISMA-ScR)

The updated database search resulted in 5355 articles. After removing 1394 duplicates, 3961 articles were screened for eligibility. An additional 24 studies from our original scoping review, and 10 studies from citation searching, were screened. *N* = 31 eligible studies were identified (Fig. 1) [22–52]. Characteristics of the included studies appear in Table 1.

**Table 1:** Characteristics of included randomised controlled trials that use CGM as a behaviour change tool (*N* = 31)

**Bibliographical data** (authors, publication year, country)	**Participants** (N; population; mean age ± SD ^a^; HbA1c eligibility (mmol/mol); insulin use ^b^; % female)	**Study duration** (weeks)	**Sensor description** (brand/model, duration)	**Sensor wear** (total days, total sensors, sensor use, duration of breaks if applicable)	**Intervention arm components** ^c,d,e^	**Comparison arm(s) components**	**Biological, Behavioural, and Psychosocial outcome(s) **^f^
Ahn et al. 2023 (Korea) [22]	*N* = 50; PreDM, T2DM; 47 ± 10y; HbA1c ≥ 39; Insulin use N/R; Female 62%	4	Abbott Freestyle Libre	Intervention and comparison: Total days: 28; Total sensors: 2; Sensor use: continuous	Unblinded CGM; Individual and group education; Dietary feedback	Unblinded CGM without guidance; Individual education	Biological: Anthropometry, Apo A/B ratio, HbA1c, HOMA-IR Behavioural: Sleep Psychological: Depression/Anxiety, Eating self-efficacy
Alfadhli et al. 2016 (Saudi Arabia) [23]	*N* = 130; GDM; 33 ± 6y; HbA1c N/R; Insulin use mixed; Female 100%	14	Medtronic Guardian REAL-Time	Total days: 7; Total sensors: 1; Sensor use: once	Unblinded CGM with prospective and retrospective CGM-based guidance on treatment plans; Concurrent glucometer use; Glucose, medication, and PA tracking	Glucometer	Biological: *HbA1c, *Pregnancy outcomes, Mean glucose, Postprandial glucose, Glucose SD, Fasting glucose, AUC for hyper- and hypoglycemia, Medication dosage, Medication need
Allen et al. 2008 (USA) [24]	*N* = 52; T2DM; 57 ± 14y; HbA1c > 58; Insulin use no; Female 52%	8	Medtronic Minimed	Total days: 3; Total sensors: 1; Sensor use: once	Unblinded CGM with retrospective CGM-based guidance on activity; Individual education	Individual education	Biological: Anthropometry, Blood pressure, HbA1c Behavioural: PA level Psychosocial: *PA self-efficacy
Allen et al. 2011 (USA) [25]	*N* = 29; T2DM; 53y; HbA1c > 53; Insulin use no; Female 100%	12	Brand and model unspecified	Unclear	CGM (blinding unclear) with retrospective CGM-based guidance on activity; Individual education; Diet, medication, and PA tracking; PA prescription; Problem-solving skills	CGM (blinding unclear, with feedback after wear); Individual education; Diet, medication, PA, and stress tracking; CGM-based advice	Biological: Anthropometry, Blood pressure, HbA1c Behavioural: Diabetes self-care, Diet, PA level Psychosocial: Depression/Anxiety, PA self-efficacy, Problem-solving skills Other: *Feasibility, Acceptability
Aronson et al. 2023 (Canada) [26]	*N* = 116; T2DM; 58 ± 10y; HbA1c ≥ 58; Insulin use no; Female 36%	16	Abbott Freestyle Libre	Total days: 98; Total sensors: 7; Sensor use: continuous	Unblinded CGM with prospective and retrospective CGM-based guidance on diet and PA changes; Individual education	Glucometer; Individual education	Biological: *TIR, Anthropometry, Glucose CV, HbA1c, Hypoglycemia, Insulin/medication need, Mean glucose, Glucose SD, TBR, TAR Other: Feasibility/Acceptability
Chekima et al. 2022 (Malaysia) [27]	*N* = 40; O/O; 26 ± 5y; HbA1c N/R; Female 58%	8	Abbott Freestyle Libre	Total days: 28; Total sensors: 2; Sensor use: intermittent (4 weeks between wears)	Unblinded CGM with prospective CGM-based guidance on diet changes; CGM training; Individual education	Individual education	Biological: *Anthropometry, Fasting glucose, Fasting insulin, HbA1c, HOMA-IR, Lipids Behavioural: Diet, PA level Other: Glycemic index knowledge
Chekima et al. 2022 (Malaysia) [28]	*N* = 14; O/O; 23 ± 5y; HbA1c N/R; Female 36%	13	Abbott Freestyle Libre	Intervention and comparison: Total days: 14; Total sensors: 1; Sensor use: once	Unblinded CGM; Individual education; High-GI meal	Arm 2: Unblinded CGM with retrospective CGM-based guidance on diet changes; Individual education; low-GI meal Arm 3: Unblinded CGM with retrospective CGM-based guidance on diet changes; Individual education; Moderate-GI meal	Biological: Glucose CV, Mean glucose, TIR, TBR, TAR Behavioural: *Diet, Meal preference
Choe et al. 2022 (Korea) [29]	*N* = 126; T2DM; 58 ± 12y; HbA1c = 53–86; Insulin use mixed; Female 40%	12	Abbott Freestyle Libre	Total days: 98; Total sensors: 7; Sensor use: continuous	Unblinded CGM with prospective CGM-based guidance on diet changes; CGM training; Individual education	Individual education, Glucose tracking, Glucometer	Biological: *HbA1c, Anthropometry, Blood pressure, Glucose CV, Fasting glucose, Lipids, TIR Behavioural: Diabetes self-care
Cosson et al. 2009 (France) [30]	*N* = 48; T1DM and T2DM; 57 ± 5y; HbA1c = 64–91; Insulin use mixed; Female 38%	13	A·Menarini Diagnostics Glucoday	Total days: 2; Total sensors: 1; Sensor use: once	Blinded CGM with retrospective CGM-based guidance on diet, activity, and medication changes	Blinded CGM (without feedback), Glucometer-based advice, Glucometer	Biological: *HbA1c, Mean glucose, Glucose SD, Minimum–maximum glucose, Frequency of hyper- and hypoglycemic excursions, LBGI, MAGE, M-value, TIR Other: Feasibility/Acceptability
Cox et al. 2020 (USA) [31]	*N* = 178; T2DM; 58 ± 12y; HbA1c ≥ 51; Insulin use no; Female 58%	13	Dexcom G5	Total days: 35; Total sensors: 5; Sensor use: intermittent (0–8 weeks between wears)	Unblinded CGM with prospective CGM-based guidance on diet and activity; CGM alarms; Diet, Glucose, and PA tracking; Group education; Glucometer between and after CGM wears	Arm 2: Diet, glucose and PA tracking; Group education, Glucometer-based advice, Glucometer Arm 3: Diet and PA tracking, Group education (focused glycaemic excursion minimization) Arm 4: Diet and PA tracking, Group education (focused on weight loss)	Biological: *HbA1c, CVD risk, Anthropometry, Lipids Behavioural: Diet, PA level Psychosocial: Diabetes distress, Diabetes empowerment, Depression/Anxiety
Furler et al. 2020 (Australia) [32]	*N* = 299; T2DM; 60 ± 10y; HbA1c > 58; Insulin use mixed; Female 41%	52	Abbott Freestyle Libre Pro	Total days: 70; Total sensors: 5; Sensor use: intermittent (11 weeks between wears)	Blinded CGM with retrospective CGM-based guidance on treatment plans; CGM training	Blinded CGM (without feedback), Usual care	Biological: *HbA1c, TIR Psychosocial: Diabetes distress
Guo et al. 2023 (China) [33]	*N* = 68; T2DM; 55 ± 14y; HbA1c N/R; Insulin use unspecified; Female 39%	4	Brand and model unspecified	Total days: 28; Total sensors: 2; Sensor use: continuous	Unblinded CGM with real-time CGM-based guidance on diet and PA; CGM training; Diet, PA, and weight tracking; Individual education; Use of a mobile app	Individual education	Biological: *HbA1c, Anthropometry, Fasting glucose, Postprandial 2-h blood glucose Behavioural: Diabetes self-care Psychosocial: Quality of Life
Haak et al. 2017 (France, Germany, UK) [34]	*N* = 224; T2DM; 59 ± 10y; HbA1c = 58–108; Insulin use yes; Female 33%	26	Abbott Freestyle Libre	Total days: 182; Total sensors: 13; Sensor use: continuous	Unblinded CGM with retrospective CGM-based guidance on diet, medication, and PA	Glucometer	Biological: *HbA1c, Anthropometry, Blood pressure, Hyperglycemia, Hypoglycemia, Insulin/medication dosage, Lipids, Mean glucose, TIR Behavioural: CGM and glucometer use frequency Other: Hospital admissions
Jospe et al. 2020 (New Zealand) [35]	*N* = 40; O/O; 42 ± 13y; HbA1c N/R; Female 55%	26	Abbott Freestyle Libre	Total days: 28; Total sensors: 2; Sensor use: continuous	Unblinded CGM with prospective CGM-based guidance on diet; CGM training; Diet tracking	Diet tracking, Individual education, Glucometer	Biological: Anthropometry, HbA1c Behavioural: Diet Psychosocial: Depression/Anxiety, Quality of Life Other: *Feasibility, Acceptability
Lee et al. 2022 (Korea) [36]	*N* = 36; T1DM; 44 ± 13y; HbA1c ≥ 53; Insulin use yes; Female 53%	12	Abbott Freestyle Libre	Intervention and comparison: Total days: 84; Total sensors: 6; Sensor use: continuous	Unblinded CGM with retrospective CGM-based guidance on diet, medication, and PA; CGM training; Individual education	Unblinded CGM without guidance; CGM training	Biological: *HbA1c, Anthropometry, Glucose CV, Hyperglycemia, Hypoglycemia, Insulin/medication dosage, Mean glucose, TIR, TBR, TAR Behavioural: Diet, PA level, CGM use frequency Other: Treatment satisfaction, Perceived hyperglycemia, Perceived hypoglycemia
Lee et al. 2023 (Korea) [37]	*N* = 294; T2DM; 56 ± 8y; HbA1c = 53–69; Insulin use no; Female 34%	48	Dexcom G5	Total days: 28; Total sensors: 4; Sensor use: intermittent (11 weeks between wears)	Unblinded CGM with real-time CGM-based guidance on diet; Blood pressure, diet, PA, and weight tracking; Concurrent glucometer use; Use of an integrated health care platform	Arm 2: Blood pressure, diet, PA, and weight tracking; use of an integrated health care platform Arm 3: Usual care	Biological: *HbA1c, Anthropometry, Glucose CV, Fasting glucose, Hypoglycemia, Lipids, Mean glucose, Glucose SD, TIR, TBR, TAR Behavioural: Number of education sessions attended Psychosocial: Diabetes treatment satisfaction Other: *Feasibility, Acceptability
Meisenhelder-Smith, 2006 (USA) [38]	*N* = 159; T2DM; 53 ± 11y; HbA1c = 53–119; Insulin use mixed; Female 55%	24	Medtronic Minimed	Total days: 3; Total sensors: 1; Sensor use: once	Unblinded CGM retrospective CGM-based guidance on diet, activity, and medication changes; Diet, glucose, medication, and PA tracking; Individual education	Individual education	Biological: *HbA1c Behavioural: Diabetes self-care Psychosocial: Health beliefs
Murphy et al. 2008 (UK) [39]	*N* = 71; PGDM; 31 ± 6y; HbA1c N/R; Insulin use mixed; Female 100%	34	Medtronic CGMS System Gold	Total days: 42; Total sensors: 6; Sensor use: intermittent (3 weeks between sensor wears)	Blinded CGM with retrospective CGM-based guidance on diet, activity, and medication changes	Usual care	Biological: *HbA1c, Pregnancy outcomes
Price et al. 2021 (USA) [40]	*N* = 70; T2DM; 60 ± 11y; HbA1c = 62–91; Insulin use no; Female 47%	12	Dexcom G6	Total days: 30; Total sensors: 3; Sensor use: intermittent (2·6 weeks between sensor wears)	Unblinded CGM with prospective and retrospective CGM-based guidance on lifestyle changes; Diet and glucose tracking; Individual education	Individual education, Glucometer	Biological: *HbA1c, TIR
Ruissen et al. 2023 (Netherlands, Spain) [41]	*N* = 226; T1DM and T2DM; 51 ± 12y; HbA1c N/R; Insulin use mixed; Female 36%	37	Abbott Freestyle Libre	Total days: 28; Total sensors: 2; Sensor use: intermittent (8 weeks between sensor wears)	Unblinded CGM; Diet, glucose, medication, mood, PA, and weight tracking; Individual education; Web and mobile app	Usual care	Biological: *HbA1c, Anthropometry, Hypoglycemia, Lipids, MAGE, Mean glucose, MODD, Glucose SD, TIR, TBR, TAR, LAGE, CVD risk, kidney disease risk, major outcomes T1DM Behavioural: Diabetes self-care, Medication adherence, PA level, Frequency SMBG Psychosocial: Diabetes distress, Quality of Life, Stress Other: Technology acceptance, Hypoglycemia awareness, Cost-effectiveness
Sato et al. 2016 (Japan) [42]	*N* = 34; T2DM; 60 ± 9y; HbA1c = 52–97; Insulin use yes; Female 41%	34	Medtronic iPro2	Total days: 15; Total sensors: 3; Sensor use: intermittent (15 weeks between sensor wears)	Blinded CGM with retrospective CGM-based guidance on lifestyle changes; Diet tracking	Blinded CGM (without feedback), Diet tracking, Glucometer	Biological: *HbA1c Other: Diabetes treatment satisfaction
Schembre et al. 2022 (USA) [43]	*N* = 50; O/O; 60 ± 5y; HbA1c < 53; Female 100%	16	Abbott Freestyle Libre	Total days: 20; Total sensors: 2; Sensor use: continuous	Unblinded CGM with prospective CGM-based guidance on diet; Individual education; Weight tracking; Group exercise classes	Individual education, Weight tracking, Group exercise classes	Biological: Adiponectin, Anthropometry, CRP, Fasting glucose, Fasting insulin, HbA1c, HOMA-IR, IGF-1, IGF-2, IGFBP-2, Lipids Other: *Feasibility, Acceptability,
Taylor et al. 2019 (Australia) [44]	*N* = 20; T2DM; 61 ± 8y; HbA1c = 41–52; Insulin use N/R; Female 50%	12	Medtronic Guardian Connect	Total days: 90; Total sensors: 9; Sensor use: continuous	Unblinded CGM with prospective CGM-based guidance on diet and activity; Concurrent glucometer use; CGM training; Diet, glucose, and PA tracking; Individual education; Diet prescription; PA prescription	Blinded CGM (without feedback), Diet, glucose, and PA tracking; Individual education; Glucometer; Diet prescription; PA prescription	Biological: *HbA1c, Anthropometry, Blood pressure, Glucose SD, CONGA, Fasting glucose, Fasting insulin, Lipids, MAGE, Medication dosage, Medication need, TIR Behavioural: Diet, Sleep, PA level Psychosocial: Depression/Anxiety, Diabetes distress Other: Feasibility/Acceptability
Tumminia et al. 2021 (Italy) [45]	*N* = 40; PGDM; 31 ± 7y; HbA1c > 48; Insulin use mixed; Female 100%	36	Intervention and comparison: Brand and model unspecified	Intervention: Total days: 252; Total sensors: 18; Sensor use: intermittent (5 weeks between sensor wears) Comparison: Total days: 42; Total sensors: 3; Sensor use: intermittent (11–17 weeks between sensor wears)	Unblinded CGM with retrospective CGM-based guidance on treatment plans; CGM training; Individual education	Unblinded CGM (with feedback after wear), CGM training, Glucometer	Biological: *HbA1c, Blood pressure, Glucose CV, Glucose SD, Mean glucose, CONGA, Diabetic neuropathy, Diabetic retinopathy, MAGE, MODD, Pregnancy outcomes, TIR, TAR, TBR Behavioural: Smoking habits
Voormolen et al. 2018 (The Netherlands) [46]	*N* = 300; GDM and PGDM; 33y; HbA1c N/R; Insulin use yes; Female 100%	24	Medtronic iPro2	Total days: 28; Total sensors: 4; Sensor use: intermittent (5 weeks between sensor wears)	Blinded CGM with retrospective CGM-based guidance on diet and activity; Concurrent glucometer use	Glucometer	Biological: *Pregnancy outcomes, Blood pressure, HbA1c, HELLP syndrome, Severe hypoglycaemia occurrence
Wada et al. 2020 (Japan) [47]	*N* = 100; T2DM; 58 ± 10y; HbA1c = 58–69; Insulin use no; Female 31%	24	Abbott Freestyle Libre	Total days: 84; Total sensors: 6; Sensor use: continuous	Unblinded CGM with prospective CGM-based guidance on diet and lifestyle changes; CGM training; Individual education	Blinded CGM (without feedback), Individual education, Glucometer	Biological: *HbA1c, Anthropometry, Blood glucose risk index, Blood pressure, Glucose SD, CONGA, Glucose CV, Fasting glucose, Lipids, MAGE, Mean glucose, Medication dosage, MODD, TIR, Uric acid, Urinary albumin Other: Diabetes treatment satisfaction
Yan et al. 2022 (China) [48]	*N* = 203; T2DM; 61y; HbA1c ≥ 53; Insulin use yes; Female 30%	13	Intervention: Abbott Freestyle Libre Comparison: Abbott Freestyle Libre Pro	Intervention and comparison: Total days: 28; Total sensors: 2; Sensor use: intermittent (4·4 weeks between sensor wears)	Unblinded CGM with prospective and retrospective CGM-based guidance on medication changes; Diet and PA tracking; Individual education	Blinded CGM (with feedback after wear), Diet and PA tracking, Individual education	Biological: *TIR, C-peptide, Mean glucose, Glucose CV, Glucose SD, HbA1c, Medication dosage, TAB, TBR Behavioural: Diet, PA level
Yeoh et al. 2018 (Singapore) [49]	*N* = 30; T2DM; 63 ± 10y; HbA1c > 64; Insulin use mixed; Female 57%	12	Medtronic iPro	Total days: 14; Total sensors: 2; Sensor use: intermittent (5 weeks between sensor wears)	Blinded CGM with retrospective CGM-based guidance on medication and lifestyle changes; Diet and PA tracking	Glucose tracking, Glucometer	Biological: *HbA1c, TIR, TAB, TBR
Yoo et al. 2008 (Korea) [50]	*N* = 65; T2DM; 55 ± 9y; HbA1c = 64–86; Insulin use mixed; Female 58%	13	Medtronic Minimed	Total days: 9; Total sensors: 3; Sensor use: intermittent (4 weeks between sensor wears)	Unblinded CGM with prospective and retrospective CGM-based guidance on diet and activity; CGM alarms, Individual education	Individual education, Glucometer-based advice, Glucometer	Biological: *HbA1c, Anthropometry, Fasting glucose, Lipids, Postprandial glucose Behavioural: Diet, PA level
Zhang et al. 2021 (China) [51]	*N* = 110; GDM; 32 ± 4y; HbA1c N/R; Insulin use no; Female 100%	2	Abbott (model unspecified)	Total days: 14; Total sensors: 1; Sensor use: once	Unblinded CGM with prospective and retrospective CGM-based guidance on diet and medication changes; Diet, medication, PA and hypoglycaemia tracking	Glucometer-based advice, Glucometer	Biological: *Hypoglycemia incidence, Anthropometry Behavioural: Diet, Glucose monitoring compliance, PA level, Regular checkups compliance, Weight monitoring compliance
Zhang et al. 2021 (China) [52]	*N* = 146; T1DM; 37 ± 20y; HbA1c ≥ 53; Insulin use yes; Female 56%	50	Brand and model unspecified	Total days: 42; Total sensors: 3; Sensor use: intermittent (22 weeks between sensor wears)	Unblinded CGM with retrospective CGM-based guidance on treatment plans; Concurrent glucometer use; CGM training	Blinded CGM (without feedback), Glucometer-based advice, Glucometer	Biological: *HbA1c, Diabetic ketoacidosis, Hypoglycaemia duration, TIR

*CGM* continuous glucose monitoring, *CONGA* continuous overlapping net glycaemic action, *CRP* c-reactive protein, *CV* coefficient of variation, *CVD* cardiovascular disease, *GDM* gestational diabetes mellitus, *HbA1c* haemoglobin A1c, *HELLP* hemolysis, elevated liver enzymes and low platelets, *LBGI* low and high blood glucose indices, *MAGE* mean amplitude of glycaemic excursions, *MODD* mean of daily difference, *N/R* not reported, *O/O* overweight/obesity, *PA* physical activity, PreDM prediabetes mellitus, *PGDM* pregestational diabetes mellitus, *SD* standard deviation of mean glucose, *T1DM* type 1 diabetes mellitus, *T2DM* type 2 diabetes mellitus, *TAR* time above range, *TBR* time below range, *TIR* time in range

^*^Denotes the primary outcome

^a^Where age was only reported for the intervention and comparison groups separately, the age of the intervention group is used

^b^Insulin use is defined as “mixed” if both participants who did and did not use insulin were included. Where insulin use was not reported, N/R is used

^c^Individual and group education was health-related education that was provided independent of CGM training and results

^d^Glucometer was only extracted if it was included as an intervention component, independent of being used for CGM calibration

^e^CGM with “prospective” or “retrospective” CGM-based guidance refers to personalised behavioural advice based on CGM trend data (e.g., diet, physical activity, medication dosage/need)

^f^Anthropometry includes measurements of body composition, body mass index, waist circumference, and/or weight

### Characteristics of CGM-based health behaviour RCTs

**Fig. 2:**
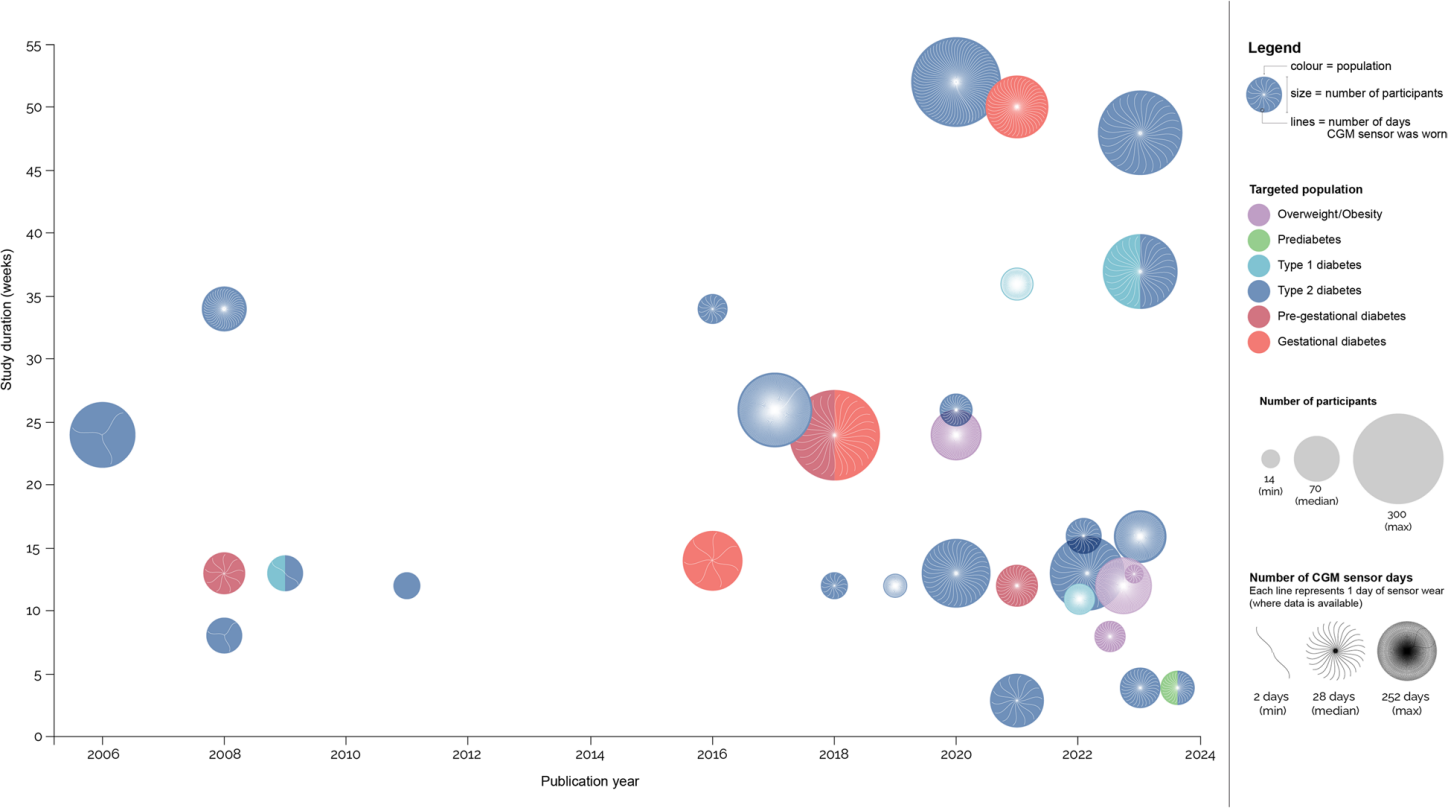
Overview of CGM-based health behaviour RCTs: study duration, targeted population, and number of sensor days (2006–2024). This figure illustrates that CGM-based health behaviour RCTs are increasing in frequency, duration, and number of days participants were asked to wear CGM sensors from 2006 to 2024. Since 2020, the target population has started to include participants without diabetes

Included RCTs were conducted in 14 countries across 4 continents with the United States being the most frequently cited location (*n* = 6/31, 19%), followed closely by South Korea (*n* = 5/31, 16%). As displayed in Fig. 2, the first included RCT was published in 2006, with almost half of the RCTs (*n* = 15/31, 48%) being published in the most recent three years (2021–2023). Included studies ranged in duration from 2–52 weeks (median 13 weeks, IQR 12–26). Most of the studies were two arm RCTs (*n* = 20/31, 90%), with two 3-arm studies (*n* = 2/31, 7%) and one 4-arm study (*n* = 1/31, 3%). The total number of study participants ranged from *N* = 14–300 (median 70, IQR 40–149).

### Characteristics of the targeted populations

Out of the 31 studies, a majority (*n* = 20, 65%) included people with type 2 diabetes (T2DM). The remaining studies included people with pre-gestational or gestational diabetes (*n* = 6/31, 19%), type 1 diabetes (T1DM) (*n* = 4/31, 13%), overweight or obesity (without diabetes) (*n* = 4/31, 13%), and/or prediabetes (*n* = 1/31, 3%). Insulin use among study participants was mixed with *n* = 10/31 (32%) studies including both insulin users and non-users, *n* = 8/31 (26%) studies exclusively included non-insulin users, *n* = 6/31 (19%) exclusively included insulin users, and *n* = 3/31 (10%) studies did not specify participants' insulin use.

### Design of health behaviour change interventions incorporating CGM

Targeted health behaviours were dietary intake (*n* = 27/31, 87%), physical activity (*n* = 16/31, 52%), and/or unspecified healthy lifestyle changes (*n* = 2/31, 6%). All the included studies were complex interventions (i.e., included multiple components) incorporating other behaviour change strategies in addition to CGM (*n* = 31/31, 100%). For example, one additional component present in most CGM-interventions was guidance (*n* = 28/31, 90%), delivered prospectively, in-real time, or retrospectively by a professional (diabetes educator (*n* = 7/28, 25%), researcher (*n* = 6/28, 21%), general healthcare provider (*n* = 5/28, 18%), healthcare specialist (*n* = 5/28, 18%), or unspecified provider (*n* = 5/28, 18%)) based on reviewing the participants’ CGM data. Prospective CGM-based guidance took place prior to the participants’ CGM wear period and involved a professional instructing participants on how to use their CGM glucose values to inform personalised dietary and physical activity changes. Real-time CGM-based guidance occurred during the CGM wear period. It used data generated from the CGM combined with physiological and/or behavioural data to generate intervention messages. Retrospective CGM-based guidance occurred after the CGM wear period, and involved a professional providing personalised recommendations for diet, physical activity, or unspecified therapy changes based on the participant’s CGM glucose values. In *n* = 6/31 (19%) of studies, participants received both prospective and retrospective CGM-based guidance. Most often (*n* = 19/31, 61%), participants received retrospective CGM-based guidance, while in *n* = 13/31 (42%), participants received prospective CGM-based guidance. In *n* = 3/13 (23%) of these studies, participants were instructed by a professional to follow a simple algorithm to make dietary or meal timing decisions based on the CGM-provided information. In two studies (*n* = 2/31, 6%) participants received real-time advice based on their CGM data. In the intervention arms, CGM was often combined with other intervention components that included health-related education (individual or group) (*n* = 20/31, 65%), diet tracking (*n* = 15/31, 48%), physical activity tracking (*n* = 11/31, 35%), and/or medication tracking (*n* = 5/31, 16%).

The comparison arms (N = 35) commonly included health-related education (*n* = 20/35, 57%), the use of a glucometer (*n* = 19/35, 54%), and/or diet tracking (*n* = 9/35, 26%). In seven comparison arms (*n *= 7/35, 20%), participants wore a CGM and received biological feedback; the distinguishing factors between the intervention and comparison arms were either the additional intervention components that were offered alongside CGM, and when the biological feedback was delivered (i.e., in real-time versus retrospectively). One study was a three-arm crossover trial, where all participants received 14 days of unblinded CGM and were randomised based on the order in which they consumed three standardised mixed dishes, varying in glycemic indices [28].

### Characteristics of CGM device and wear

CGM manufacturer was specified in most studies (*n* = 27/31, 87%). Abbott (*n* = 14/31, 45%) was most frequently used, followed by Medtronic (*n* = 9/31, 29%), Dexcom (*n* = 3/31, 10%), and A. Menarini Diagnostics (*n* = 1/31, 3%). The Abbott Freestyle Libre (*n* = 12/31, 39%) was the most commonly used model of CGM. Single CGM wears ranged from 2–14 days in duration, depending on the manufacturer (Medtronic = 2–10 day wears, Abbott = 10–14 day wears, Dexcom = 7–10 day wears). Across the reporting studies (*n* = 30/31, 97%), the number of sensors worn ranged from 1–18 (median 3 wears, IQR 2–6), which resulted in a total number of CGM wear days of 2–252 days per intervention (median 28 days, IQR 14–63). For studies with multiple CGM wears (*n* = 24/30, 80%), CGM was worn continuously during the intervention in *n* = 11/24 (46%) studies; whereas, in the other *n* = 13/24 (54%) studies, participants wore CGM intermittently (median 3 wears, IQR 2–4) with breaks between wears (median 5 weeks, IQR 4–11).

### Communication of CGM-based biological feedback

**Fig. 3:**
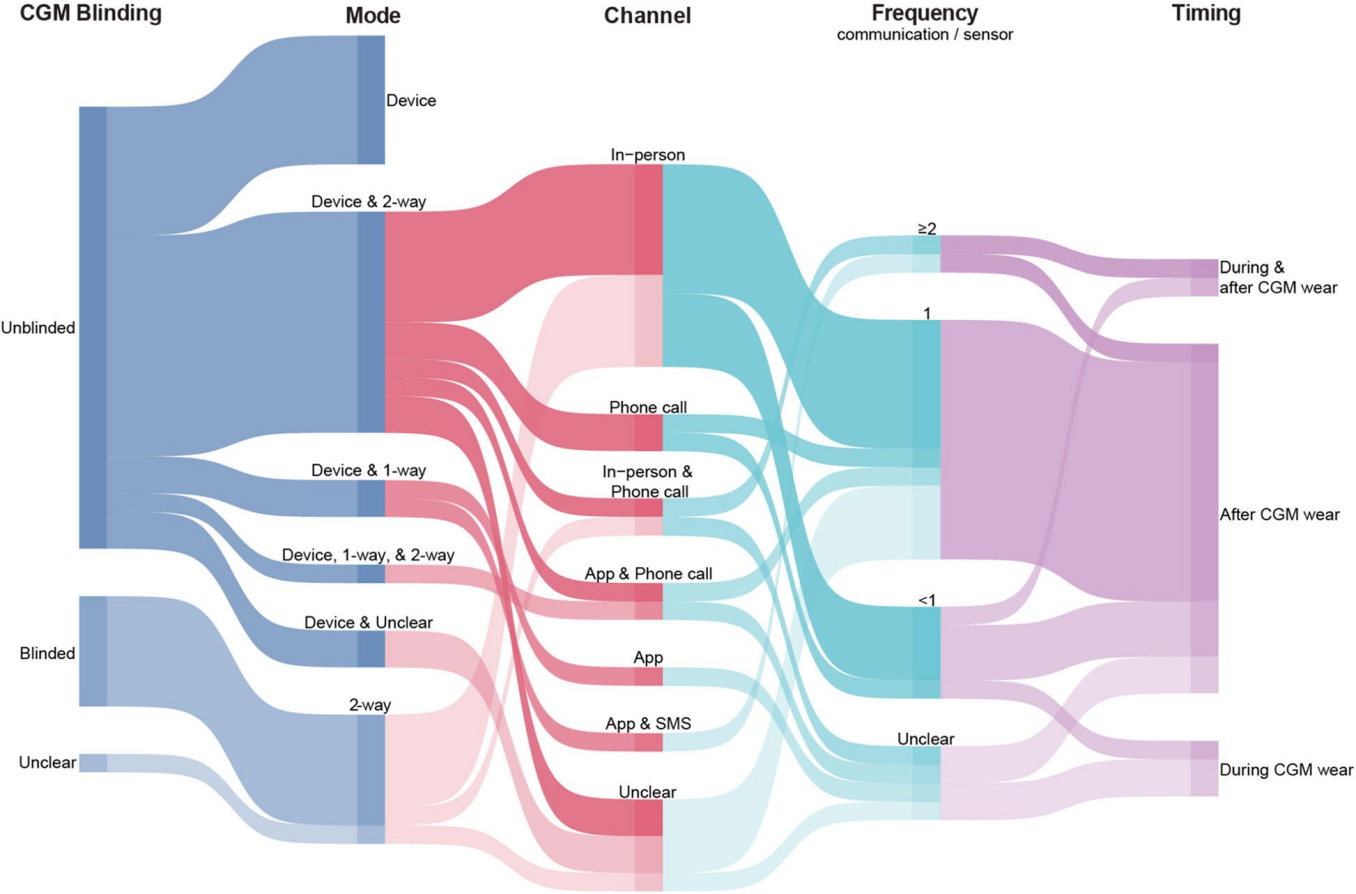
Delivery of CGM-based biological feedback in behaviour change interventions l (*N* = 31). This figure illustrates how studies delivered CGM-based biological feedback. The size of the band indicates the number of studies. “CGM blinding” describes whether CGM data were visible (unblinded) or were not visible (blinded) to a study participant in real-time during the CGM wear period(s). “Mode”, “Channel”, “Frequency”, and “Timing” are specific to how CGM-based biological feedback was communicated. “Frequency” was calculated by the number of one- or two-way feedback sessions divided by the number of sensors worn. “Unclear” was used when the study protocols did not provide related information. From this figure we can see that the plurality of studies used unblinded CGM, with device and two-way communication, which was usually in-person, at a frequency of 1 communication session per CGM sensor, which was provided after CGM wear

The communication of CGM-based biological feedback varied by whether CGM data were made visible (“unblinded”) or not visible (“blinded”) to participants during the CGM wear(s), and whether one-way (e.g., via one-way email) or two-way (e.g., via in-person discussion) delivery of CGM-based biological feedback was provided (Fig. 3). There were 3 predominant forms of communication: (1) via *unblinded* CGM device *with* one- or two-way communication (*n* = 17/31, 55%); (2) via *blinded* CGM device *with* one- or two-way communication (*n* = 6/31, 19%); and (3) via *unblinded* CGM device *without* one- or two-way communication (*n* = 7/31, 23%). One study was unclear about blinding but did provide two-way communication.

There was variability—and occasionally a lack of clarity—in how the feedback was conveyed to participants in terms of the mode, channel, frequency, and timing. Most commonly, when reported, CGM-based biological feedback was provided by the mode of CGM device and two-way communication (*n* = 12/31, 39%), through two-way communication alone (*n* = 7/31, 23%) or device alone (*n* = 7/31, 23%). Two-way communication was most often delivered in-person (*n* = 13/31, 42%) and/or over the phone (*n* = 6/31, 19%), and typically occurred after CGM wear (*n* = 19/31, 61%), once per CGM wear (*n* = 13/31; 42%). All feedback for one- and two-way communication was delivered by a human, as opposed to automated feedback (digital or artificial intelligence).

### Targeted biological, behavioural and psychosocial outcomes

Multiple biological, behavioural, and psychosocial outcomes were reported in the included RCTs (Table 1). Biological outcomes were reported by all included studies and were often the primary outcome(s) (*n* = 25/31, 81%). Change in HbA1c was reported as an outcome in a majority of studies (*n *= 29/31, 94%). Other commonly reported biological outcomes were anthropometry (*n* = 18/31, 58%), time in range (*n *=16/31, 52%), hypoglycemia (*n* = 15/31, 48%), mean glucose (*n* = 11/31, 35%), lipids (*n* = 10/31, 32%), standard deviation of mean glucose (*n* = 9, 29%), and fasting glucose (*n* = 9/31, 29%). Seventeen studies (55%) included behavioural outcomes, which were most frequently diet (*n* = 11/35, 32%), physical activity (*n* = 10/31, 32%), and diabetes self-care (*n* = 5/31, 16%). Eight studies (*n* = 8/31, 26%) included psychosocial outcomes, including depression/anxiety (*n* = 6/31, 19%), and diabetes distress (*n* = 4/31, 13%). Six studies (19%) included intervention feasibility and acceptability as an outcome.

## Discussion

As we enter the precision health era, biosensors like CGM exemplify how biological feedback can potentially revolutionise health behaviour change interventions. To our knowledge, this is the first review to comprehensively explore the characteristics of CGM-based interventions that use biological feedback to support health behaviour change. We found that a significant portion of the included studies were published recently, with nearly half (*N* = 15/31, 48%) published within the last 3 years, indicating considerable growth of the CGM evidence base. Most studies involved people with T2DM and assessed HbA1c as an outcome. All were complex, multi-component interventions, often combining CGM with prospective or retrospective guidance; health-related education; and diet, physical activity, or medication tracking. CGM-based biological feedback was often delivered through in-person discussions after wearing CGM. These detailed understandings of CGM interventions—how they were operationalized, what they involved and what they targeted, alone and in combination with other behaviour change components—is an important first step to systematically understanding the relationship of these various elements with intervention effects.

The first objective of this review was to provide an overview of patient populations, health behaviours, and health-related outcomes associated with CGM-based biological feedback interventions. We found a lack of RCTs investigating the benefits of using CGM for behaviour change among individuals without diabetes, despite interests in this application of the technology in the digital health market. Nevertheless, research in this area appears to be on the rise, with four RCTs investigating the use of CGM-based biological feedback in individuals without diabetes since 2020, and one RCT including individuals with prediabetes published in 2023. CGM interventions primarily targeted diet and physical activity, aligning with general biological feedback [6], and precision health interventions [53]. Most interventions assessed HbA1c as an intervention outcome, likely due to the prevalence of diabetes in the studies. Future research should explore CGM's impact on other health biomarkers (e.g., weight, CVD risk factors), potentially benefiting individuals without diabetes. This research could provide a scientific basis for the goals of digital health startups focusing on outcomes like weight loss and chronic disease prevention.

The second objective of this review was to describe how CGM is used in biological feedback interventions. In most of the reviewed RCTs, CGM-measured glucose levels were used as input to generate guidance to improve healthy lifestyle behaviours, often through retrospective feedback by professionals on diet, activity, or disease management plans. However, there was considerable variation in how CGM-based feedback was delivered to participants, including differences in mode, channel, frequency, and timing. The noted variability in communication has been observed previously in another context [54] and may vary depending on the population, biomarker, and targeted outcome [6, 55]. More recent studies have provided CGM-based biological feedback from an unblinded CGM over longer durations, and have incorporated the use of one-way communication (e.g., via a mobile app). Nevertheless, the delivery of CGM-based guidance was mainly reliant on human interaction versus artificial intelligence. Consistent with precision health literature [53], a majority of personalised feedback in the present review relied on human interaction for developing and communicating CGM-based guidance. Despite human interaction being potentially more effective in achieving health outcomes [56], limitations like cost, availability, and reach limit widespread use. This highlights a potential research gap and opportunity for more novel approaches, such as artificial intelligence, to be integrated into mobile platforms to automate the delivery of meaningful, personalised biological feedback. An example of this was showcased in a recent RCT, where Guo and colleagues instructed intervention participants with T2DM to use a mobile app, which used artificial intelligence to analyse and integrate unblinded CGM data and participant self-reported diet and activity data to provide personalised feedback on foods and exercises that were least and most beneficial for the participant’s personal glucose management [33].

The main strength of this review was our application of a systematic method to capture and characterise CGM-based biological feedback interventions in unprecedented detail. This thorough mapping provides a starting point for further examination of individual intervention components and their impact, paving the way for inventive intervention designs. However, there are limitations. Our inclusion criteria focused only on RCTs and adults, with the purpose of laying the groundwork for a future meta-analysis of study effects based on commonly targeted outcomes (e.g., HbA1c) identified through this review. Some studies lacked clarity in how CGM was used and how intervention components were implemented, which we addressed by searching for protocols, corresponding with authors, and conducting a thorough search of clinical trial registries.

To our knowledge, this is the first scoping review to describe how CGM is used within interventions that promote behaviour change. Despite the burgeoning interest in CGM and its application in the digital health market, academic evidence supporting the use of CGM-based interventions for behaviour change is mostly limited to people living with diabetes. To advance CGM-based precision health interventions, collaboration between academia and industry will be crucial. This collaboration can expedite the translation of research to real-world applications, enabling more effective data-driven interventions.

Based on the findings of this scoping review, we have identified a substantial body of literature on the effects of using CGM as a tool for biological feedback to reduce HbA1c levels. We plan to evaluate these effects in a subsequent meta-analysis (CRD42024514135). In addition to this, given the multi-component nature of these interventions, we plan to further investigate the behaviour change techniques that accompany CGM-based biological feedback interventions, with the long-term goal of identifying optimal combinations of behaviour change techniques to offer in combination with CGM to improve health outcomes (CRD42023398390). These future directions underscore the importance of our review, which serves not only as a current snapshot but also as a foundational resource for upcoming research efforts. This review has the potential to guide the design of future research to determine best practices for implementing CGM-based precision health interventions and contribute to guidelines for precision health interventions using biological feedback. Best practices can address key aspects such as the duration and frequency of sensor wear, communication of CGM data, and behaviour change techniques to deliver alongside CGM-based biological feedback. As biosensors like CGM play an expanding role in healthcare, rigorous evaluation is essential to inform public health and clinical guidelines.

## Data Availability

The dataset supporting the conclusions of this review is currently available in the Zenodo repository, 10.5281/zenodo.10822226. The dataset contains references to the included articles, as well as the data extracted for each study.

## Appendix 1

### Search strategy

Searches last conducted 1/16/2024.

### Ovid MEDLINE(R) ALL < 1946 to January 12, 2024 >

**Table:**

#	Searches	Results
1	blood glucose self-monitoring/	10,221
2	(glucose adj3 (monitor* or sensor or sensors or biosensor*)).tw	20,534
3	(CGM or CGMS or rtCGM or rt-CGM or isCGM or is-CGM).tw	4811
4	("freestyle libre" or dexcom or "guardian sensor" or eversense).tw	741
5	or/1–4	24,602
6	glucose/ or blood glucose/ or glucose.tw	663,423
7	monitoring, physiologic/ or monitoring, ambulatory/ or physiological feedback/ or monitor*.tw	1,054,480
8	6 and 7	38,715
9	5 or 8	45,853
10	behavior/	30,208
11	exp health behavior/	364,425
12	behavior control/	1937
13	behavioral medicine/	1753
14	behavioral research/	3542
15	feeding behavior/	93,482
16	health, knowledge, attitudes, practice/	127,666
17	exp healthy lifestyle/	12,289
18	exp health promotion/	86,124
19	exp motivation/	196,873
20	risk reduction behavior/	14,328
21	self-efficacy/	24,675
22	self-care/	36,327
23	self-management/	5649
24	awareness/	22,159
25	exp inhibition, psychological/	13,258
26	"Treatment Adherence and Compliance"/	1076
27	Patient Compliance/	60,760
28	patient participation/	29,715
29	public health/	97,132
30	public health practice/	5682
31	preventive medicine/	12,029
32	prevention & control.fs	1,475,302
33	preventive health services/	14,501
34	exp primary prevention/	185,762
35	secondary prevention/	22,789
36	tertiary prevention/	202
37	smoking prevention/	18,668
38	harm reduction/	4233
39	treatment outcome/ and (lifestyle/ or psychology.fs.)	57,911
40	((behavio?r* or lifestyle) adj3 (chang* or modif* or promot*)).tw	135,306
41	"health behavio?r*".tw	31,268
42	"healthy lifestyle".tw	9485
43	(self adj3 (care or management or efficacy)).tw	92,992
44	awareness.tw	210,308
45	((risk or harm or "sedentary behavio?r") adj3 reduc*).tw	198,994
46	"weight loss".tw	110,413
47	"weight control".tw	7250
48	(smok* adj3 (behavio?r* or cessation or quit*)).tw	48,004
49	"self regulat*".tw	16,165
50	(motivated or motivation).tw	122,184
51	(adherence or compliance).tw	294,290
52	(prevention or preventive).tw	819,587
53	"health promotion".tw	37,566
54	(improv* adj3 (activit* or eating or diet* or health or fitness)).tw	171,338
55	((exercise or "physical activity" or diet* or eating or weight) adj3 (behavio?r* or chang* or maint* or motivat* or promot* or modif*)).tw	158,050
56	"public health".tw	338,141
57	or/10–56	4,049,210
58	9 and 57	11,206
59	limit 58 to medline	9945
60	58 not 59	1261
61	randomized controlled trial.pt	606,715
62	controlled clinical trial.pt	95,522
63	randomi#ed.ab	753,163
64	clinical trials as topic.sh	201,604
65	randomly.ab	424,908
66	trial.ti	300,662
67	61 or 62 or 63 or 64 or 65 or 66	1,577,353
68	exp animals/ not humans.sh	5,186,087
69	67 not 68	1,459,060
70	59 and 69	2096
71	random*.tw	1,480,911
72	trial.tw	787,819
73	71 or 72	1,836,247
74	60 and 73	310
75	70 or 74	2406

### Embase.com Embase

**Table:**

No.	Query	Results
#56	#55 AND [embase]/lim	5031
#55	#53 AND #54	5515
#54	'crossover procedure':de OR 'double-blind procedure':de OR 'randomized controlled trial':de OR 'single-blind procedure':de OR random*:de,ab,ti OR factorial*:de,ab,ti OR crossover*:de,ab,ti OR ((cross NEXT/1 over*):de,ab,ti) OR placebo*:de,ab,ti OR ((doubl* NEAR/1 blind*):de,ab,ti) OR ((singl* NEAR/1 blind*):de,ab,ti) OR assign*:de,ab,ti OR allocat*:de,ab,ti OR volunteer*:de,ab,ti	3,285,960
#53	#9 AND #52	23,858
#52	#10 OR #11 OR #12 OR #13 OR #14 OR #15 OR #16 OR #17 OR #18 OR #19 OR #20 OR #21 OR #22 OR #23 OR #24 OR #25 OR #26 OR #27 OR #28 OR #29 OR #30 OR #31 OR #32 OR #33 OR #34 OR #35 OR #36 OR #37 OR #38 OR #39 OR #40 OR #41 OR #42 OR #43 OR #44 OR #45 OR #46 OR #47 OR #48 OR #49 OR #50 OR #51	5,081,329
#51	'public health':ti,ab	407,163
#50	((exercise OR 'physical activity' OR diet* OR eating OR weight) NEAR/3 (change OR behavior* OR behaviour* OR modif* OR maint* OR motivat* OR promot*)):ti,ab	166,325
#49	(improv* NEAR/3 (activit* OR eating OR diet* OR health OR fitness)):ti,ab	216,593
#48	'health promotion':ti,ab	43,932
#47	prevention:ti,ab OR preventive:ti,ab	1,105,890
#46	adherence:ti,ab OR compliance:ti,ab	459,780
#45	motivated:ti,ab OR motivation:ti,ab	145,262
#44	'self regulat*':ti,ab	18,388
#43	(smok* NEAR/3 (behavior* OR behaviour* OR cessation OR quit*)):ti,ab	62,617
#42	'weight control':ti,ab	9501
#41	'weight loss':ti,ab	184,830
#40	((risk OR harm OR 'sedentary behavior' OR 'sedentary behaviour') NEAR/3 reduc*):ti,ab	283,812
#39	awareness:ti,ab	299,294
#38	(self NEAR/3 (care OR management OR efficacy)):ti,ab	120,880
#37	'healthy lifestyle*':ti,ab	16,342
#36	'health behavior*':ti,ab OR 'health behaviour*':ti,ab	36,695
#35	((behavior* OR behaviour* OR lifestyle) NEAR/3 (change* OR modif* OR promot*)):ti,ab	171,117
#34	'treatment outcome'/de AND (psychology:de OR 'lifestyle'/de)	13,297
#33	'smoking prevention'/de	1155
#32	'tertiary prevention'/de	769
#31	'secondary prevention'/de	35,948
#30	'primary prevention'/de	47,871
#29	'prevention'/de	328,530
#28	'preventive health service'/de	32,192
#27	prevention:lnk OR 'prevention and control'/de	1,367,259
#26	'preventive medicine'/de	32,084
#25	'public health'/de	257,322
#24	'patient participation'/de	36,608
#23	'patient compliance'/exp	195,111
#22	'inhibition (psychology)'/exp	8457
#21	'awareness'/de	139,076
#20	'self care'/de	78,054
#19	'risk reduction'/de	133,664
#18	'motivation'/exp	180,581
#17	'health promotion'/de	113,029
#16	'lifestyle modification'/de	53,955
#15	'healthy lifestyle'/de	9587
#14	'feeding behavior'/de	101,348
#13	'behavior change'/de	52,586
#12	'behavior control'/de	4869
#11	'health behavior'/exp	510,888
#10	'behavior'/de	177,800
#9	#5 OR #8	94,503
#8	#6 AND #7	65,527
#7	'physiologic monitoring'/de OR 'ambulatory monitoring'/de OR monitor*:ti,ab	1,416,545
#6	'glucose blood level'/de OR 'glucose level'/de OR 'glucose'/de OR glucose:ti,ab	1,014,476
#5	#1 OR #2 OR #3 OR #4	59,497
#4	'freestyle libre':ti,ab OR dexcom:ti,ab OR 'guardian sensor':ti,ab OR eversense:ti,ab	2458
#3	cgm:ti,ab OR cgms:ti,ab OR rtcgm:ti,ab OR 'rt cgm':ti,ab OR iscgm:ti,ab OR 'is cgm':ti,ab	11,290
#2	(glucose NEAR/3 (monitor* OR sensor OR sensing OR biosensor*)):ti,ab	34,920
#1	'blood glucose monitoring'/de OR 'continuous glucose monitoring system'/de	40,017

### Cochrane Library CENTRAL

Only exported database search results not trial registers for CT.gov or ICTRP.

IDSearch

#1 MeSH descriptor: [Blood Glucose Self-Monitoring] this term only

#2 (glucose NEAR/3 (monitor* OR sensor OR sensors OR biosensor*)):ti,ab

#3 (CGM or CGMS or rtCGM or rt-CGM or isCGM or is-CGM):ti,ab

#4 ("freestyle libre" or dexcom or "guardian sensor" or eversense):ti,ab

#5 {OR #1-#4}

#6 [mh ^"glucose"] OR [mh ^"blood glucose"] OR glucose:ti,ab

#7 [mh ^"monitoring, physiologic"] OR [mh ^"monitoring, ambulatory"] OR [mh ^"physiological feedback"] OR monitor*:ti,ab

#8 #6 AND #7

#9 #5 OR #8

#10 MeSH descriptor: [Behavior] this term only

#11 MeSH descriptor: [Health Behavior] explode all trees

#12 MeSH descriptor: [Behavior Control] this term only

#13 MeSH descriptor: [Behavioral Medicine] this term only

#14 MeSH descriptor: [Behavioral Research] this term only

#15 MeSH descriptor: [Feeding Behavior] this term only

#16 MeSH descriptor: [Health Knowledge, Attitudes, Practice] this term only

#17 MeSH descriptor: [Healthy Lifestyle] explode all trees

#18 MeSH descriptor: [Health Promotion] explode all trees

#19 MeSH descriptor: [Motivation] explode all trees

#20 MeSH descriptor: [Risk Reduction Behavior] this term only

#21 MeSH descriptor: [Self Efficacy] this term only

#22 MeSH descriptor: [Self Care] this term only

#23 MeSH descriptor: [Self-Management] this term only

#24 MeSH descriptor: [Awareness] this term only

#25 MeSH descriptor: [Inhibition, Psychological] explode all trees

#26 MeSH descriptor: [Treatment Adherence and Compliance] this term only

#27 MeSH descriptor: [Patient Compliance] this term only

#28 MeSH descriptor: [Patient Participation] this term only

#29 MeSH descriptor: [Public Health] this term only

#30 MeSH descriptor: [Public Health Practice] this term only

#31 MeSH descriptor: [Preventive Medicine] this term only

#32 MeSH descriptor: [] explode all trees and with qualifier(s): [prevention & control—PC]

#33 MeSH descriptor: [Preventive Health Services] this term only

#34 MeSH descriptor: [Primary Prevention] explode all trees

#35 MeSH descriptor: [Secondary Prevention] this term only

#36 MeSH descriptor: [Tertiary Prevention] this term only

#37 MeSH descriptor: [Smoking Prevention] this term only

#38 MeSH descriptor: [Harm Reduction] this term only

#39 [mh ^"Treatment Outcome"] AND ([mh /PX] OR [mh ^Lifestyle])

#40 ((behavior* OR behaviour* OR lifestyle) NEAR/3 (change* OR modif* or promot*)):ti,ab

#41 (health NEXT (behavior* OR behaviour*)):ti,ab

#42 ("healthy lifestyle" or "healthy lifestyles"):ti,ab

#43 (self NEAR/3 (care OR management or efficacy)):ti,ab

#44 awareness:ti,ab

#45 ((risk or harm or "sedentary behavior" OR "sedentary behaviour") NEAR/3 reduc*):ti,ab

#46 "weight loss":ti,ab

#47 "weight control":ti,ab

#48 (smok* NEAR/3 (behavior* OR behaviour* OR cessation OR quit*)):ti,ab

#49 (self NEXT regulat*):ti,ab

#50 motivated:ti,ab or motivation:ti,ab

#51 adherence:ti,ab or compliance:ti,ab

#52 prevention:ti,ab OR preventive:ti,ab

#53 "health promotion":ti,ab

#54 (improv* NEAR/3 (activit* OR eating OR diet* OR health OR fitness)):ti,ab

#55 ((exercise OR "physical activity" or diet* or eating or weight) NEAR/3 (change OR behavior* OR behaviour* OR modif* or maint* or motivat* or promot*)):ti,ab

#56 "public health":ti,ab

#57 {OR #10-#56}

#58 #9 AND #57 in Trials

### EbscoHOST PsycINFO

**Table:**

#	Query	Results
S49	S44 AND S48	104
S48	S45 OR S46 OR S47	375,785
S47	TI trial OR AB trial	216,525
S46	TI random* OR AB random*	246,375
S45	(DE "Randomized Controlled Trials") OR (DE "Clinical Trials")	13,305
S44	S5 AND S43	540
S43	S6 OR S7 OR S8 OR S9 OR S10 OR S11 OR S12 OR S13 OR S14 OR S15 OR S16 OR S17 OR S18 OR S19 OR S20 OR S21 OR S22 OR S23 OR S24 OR S25 OR S26 OR S27 OR S28 OR S29 OR S30 OR S31 OR S32 OR S33 OR S34 OR S35 OR S36 OR S37 OR S38 OR S39 OR S40 OR S41 OR S42	941,252
S42	TI "public health" OR AB "public health"	62,433
S41	TI ((exercise OR "physical activity" OR diet* OR eating OR weight) N3 (behavio#r* OR chang* OR maint* OR motivat* OR promot* OR modif*)) OR AB ((exercise OR "physical activity" OR diet* OR eating OR weight) N3 (behavio#r* OR chang* OR maint* OR motivat* OR promot* OR modif*))	51,856
S40	TI ( improv* N3 (activit* OR eating OR diet* OR health OR fitness)) OR AB ( improv* N3 (activit* OR eating OR diet* OR health OR fitness))	45,920
S39	TI ( prevention OR preventive) OR AB ( prevention OR preventive)	163,470
S38	TI ( adherence OR compliance) OR AB ( adherence OR compliance)	62,024
S37	TI (motivated OR motivation) OR AB (motivated OR motivation)	147,037
S36	TI "self regulat*" OR AB "self regulat*"	25,748
S35	TI (smok* N3 (behavio#r* OR cessation or quit*)) OR AB (smok* N3 (behavio#r* OR cessation OR quit*))	22,199
S34	TI "weight control" OR AB "weight control"	2,858
S33	TI "weight loss" OR AB "weight loss"	12,914
S32	TI ((risk OR harm OR "sedentary behavio#r") N3 reduc*) OR AB ((risk OR harm OR"sedentary behavio#r") N3 reduc*)	37,405
S31	TI awareness OR AB awareness	117,191
S30	TI "healthy lifestyle" OR AB "healthy lifestyle"	2,791
S29	TI "health behavio#r*" OR AB "health behavio#r*"	19,254
S28	TI ( self N3 (care OR management OR efficacy)) OR AB ( self N3 (care OR management OR efficacy))	78,827
S27	TI ( (behavio#r* OR lifestyle) N3 (chang* OR modif* OR promot*)) OR AB ( (behavio#r* OR lifestyle) N3 (chang* OR modif* OR promot*))	89,848
S26	(DE "Treatment Outcomes") OR (DE "Health Outcomes")	47,056
S25	DE "Harm Reduction"	5,329
S24	DE "Substance Use Prevention" OR DE "Relapse Prevention"	7,800
S23	DE "Prevention"	37,700
S22	DE "Public Health Services"	3,346
S21	(DE "Public Health")	31,085
S20	DE "Compliance"	5,919
S19	DE "Treatment Compliance"	18,048
S18	(DE "Awareness") OR (DE "Health Awareness")	27,559
S17	DE "Self-Management"	8,485
S16	DE "Self-Care"	3,989
S15	DE "Self-Efficacy"	30,245
S14	DE "Health Behavior Measures"	116
S13	DE "Motivation" OR DE "Goals" OR DE "Incentives"	109,244
S12	DE "Health Promotion"	38,686
S11	DE "Lifestyle Changes"	1,574
S10	DE "Health Attitudes"	11,612
S9	DE "Behavior Modification"	10,802
S8	DE "Behavioral Medicine"	1,578
S7	DE "Health Behavior" OR DE "Health Risk Behavior" OR DE "Preventive Health Behavior"	46,321
S6	DE "Behavior"	36,774
S5	S1 OR S2 OR S3 OR S4	909
S4	TI ("freestyle libre" OR dexcom OR "guardian sensor" OR eversense) OR AB ("freestyle libre" OR dexcom OR "guardian sensor" OR eversense)	7
S3	TI (CGM OR CGMS OR rtCGM OR rt-CGM OR isCGM OR is-CGM) OR AB (CGM OR CGMS OR rtCGM OR rt-CGM OR isCGM OR is-CGM)	128
S2	TI (glucose N3 (monitor* OR sensor OR sensors OR biosensor*)) OR AB (glucose N3 (monitor* OR sensor OR sensors OR biosensor*))	815
S1	(DE "Glucose" OR DE "Blood Sugar") AND (DE "Monitoring" OR DE "Medical Therapeutic Devices")	65

### ProQuest Dissertations & Theses Global

(ti(random*) OR ab(random*) OR ti(trial) OR ab(trial)) AND ((Exact("glucose monitoring") OR ((Exact("glucose") OR ti(glucose) OR ab(glucose)) AND (ti(monitor* OR sensor OR sensors OR biosensor*) OR ab(monitor* OR sensor OR sensors OR biosensor*)))) AND (Exact("behavior" OR "health behavior" OR "behavior modification" OR "eating behavior" OR "patient compliance" OR "self awareness" OR "disease prevention" OR "compliance" OR "motivation" OR "preventive medicine" OR "health promotion" OR "public health" OR "public health health sciences" OR "prevention" OR "harm reduction") OR ti((behavior OR behaviour OR lifestyle) NEAR/3 (chang* OR modif* OR promot*)) OR ab ti((behavior OR behaviour OR lifestyle) NEAR/3 (chang* OR modif* OR promot*)) OR ti(self NEAR/3 (care OR management OR efficacy)) OR ab(self NEAR/3 (care OR management OR efficacy)) OR ti(health p/0 behavior OR health p/0 behaviour) OR ab(health p/0 behavior OR health p/0 behaviour) OR ti(“healthy lifestyle”) OR ab(“healthy lifestyle”) OR ti(awareness) OR ab(awareness) OR ti((risk OR harm OR “sedentary behavior” OR “sedentary behaviour”) NEAR/3 reduc*) OR ab((risk OR harm OR “sedentary behavior” OR “sedentary behaviour”) NEAR/3 reduc*) OR ti(“weight loss”) OR ab(“weight loss”) OR ti(“weight control”) OR ab(“weight control”) OR ti(smok* NEAR/3 (behavior OR behaviour OR cessation OR quit*)) OR ab(smok* NEAR/3 (behavior OR behaviour OR cessation OR quit*)) OR ti(("self regulate" OR "self regulated" OR "self regulating" OR "self regulation" OR "self regulatory")) OR ab(("self regulate" OR "self regulated" OR "self regulating" OR "self regulation" OR "self regulatory")) OR ti(motivated OR motivation) OR ab(motivated OR motivation) OR ti(adherence OR compliance) OR ab(adherence OR compliance) OR ti(prevention OR preventive) OR ab(prevention OR preventive) OR ti(improv* NEAR/3 (activit* OR eating OR diet* OR health OR fitness)) OR AB(improv* NEAR/3 (activit* OR eating OR diet* OR health OR fitness)) OR ti((exercise OR “physical activity” OR diet* OR eating OR weight) NEAR/3 (behavior OR behaviour OR chang* OR maint* OR motivat* OR promot* OR modif*)) OR ab((exercise OR “physical activity” OR diet* OR eating OR weight) NEAR/3 (behavior OR behaviour OR chang* OR maint* OR motivat* OR promot* OR modif*)) OR ti(“public health”) OR ab(“public health”))).

## Appendix 2

### Extracted data

1. Bibliographical data (title, authors, year of publication, location)
2. Participant characteristics (population, insulin use, number of participants, % female, mean age, age range, HbA1c eligibility criteria, baseline HbA1c)
3. Primary and secondary outcomes
4. Targeted behaviours
5. Duration of intervention
6. Description of Intervention and comparison arms
7. Detailed description of CGM use
  - Brand and model of CGM
  - Blinded versus unblinded CGM
  - Duration of CGM sensor
  - Number of CGM sensors worn
  - Duration between CGM wear sessions (if worn more than once)
  - Communication of CGM results beyond the device (if any)
  - Who provided CGM feedback (e.g., human, artificial intelligence)
  - Channel used to provide CGM feedback (e.g., in-person, app, email)
  - Frequency of CGM feedback
  - Timing of CGM feedback (e.g., during or after CGM wear)
  - What (if anything) was personalised based on CGM data (e.g., diet, physical activity)
  - CGM metrics shared or interpreted (e.g., time in range, mean glucose)
